# Individualized responses to acupuncture in premature ovarian insufficiency: A study protocol for a nested case-control trial with transcriptome analysis

**DOI:** 10.1016/j.heliyon.2024.e37859

**Published:** 2024-09-12

**Authors:** Shiyu Feng, Yu Luo, Yan Chen, Haimin Zhu, Tianqi Zhao, Fei Ma, Yanting Lin, Yan Ning, Jiaman Wu

**Affiliations:** a Department of Chinese Medicine, Shenzhen Maternity and Child Healthcare Hospital, Shenzhen 518028, China; b Guangzhou University of Chinese Medicine, Guangzhou 510405, China; c Southern Medical University, Guangzhou 510515, China

**Keywords:** Acupuncture, Premature ovarian insufficiency, Individual curative effect, Transcriptome, Protocol

## Abstract

**Background:**

Premature ovarian insufficiency (POI), a modifiable cause of infertility with substantial implications for women's well-being, prompts the exploration of efficacious adjunctive treatments. Acupuncture emerges as a promising therapeutic avenue; however, the nuanced effects of acupuncture in POI warrant more comprehensive investigation. The intricate mechanisms dictating individualized responses remain elusive. This trial seeks to assess the effectiveness of acupuncture as an adjunctive treatment for POI, concurrently delving into the impact of transcriptome analysis on peripheral blood to unravel the underpinnings of these individual variations. The overarching objective is to enrich our comprehension of acupuncture's therapeutic potential in the context of POI, with a view to advancing holistic patient care.

**Methods/design:**

Constituting an open-label, nested case-control study, this research endeavors to enroll 108 women diagnosed with POI. Participants will be randomly assigned in a 1:1 ratio to either the study group or the control group, each comprising 54 subjects. Ten patients from each group meeting specific criteria will partake in transcriptome analysis. An additional 10 subjects meeting the study criteria will form a healthy control group. The study group will exclusively undergo acupuncture treatment, while the control group will solely receive Fenmutong. Acupuncture sessions, administered thrice weekly across three menstrual cycles from the fifth day of menstruation, constitute the intervention. Primary outcome measurement rests on Follicle-Stimulating Hormone (FSH) levels, supplemented by secondary assessments encompassing biometric features, hormone biomarkers, anxiety and depression scores, and transcriptome analysis. Baseline measurements precede intervention, with post-intervention evaluations following. The study endeavors to discern specific genes linked to individualized responses to acupuncture. Data analysis, employing SPSS 25.0 software, incorporates a meticulous examination of peripheral blood samples for transcriptome analysis. The investigation aspires to shed light on genetic determinants influencing the effects of acupuncture on women with POI, thereby fostering elevated standards in patient care and management.

**Discussion:**

This study pivots on the principal objective of scrutinizing the efficacy of acupuncture as an adjunctive treatment for POI. Beyond effectiveness, it undertakes an exploration of the intricate mechanisms underlying the diverse responses exhibited by individuals in the context of acupuncture, augmenting the depth of understanding in this therapeutic domain.

## 1 Strengths and limitations of this study

This protocol aims to provide patients, clinical practitioners and policy makers with more evidence on the efficacy and safety of acupuncture in the treatment of 108 POIs.

The data extraction and management, assessment of risk of bias sections will be carried out by two or more researchers independently.

The non-inclusion of studies published in languages other than English and Chinese may result in limitations related to publication bias.

Multiple types of acupuncture therapies may increase the risk of heterogeneity.

## 2 Trial Registration

China Clinical Trial Registration Centre (No. ChiCTR2300068981). Clinical trial registry by referring to https://www.chictr.org.cn/hvshowproject.html?id=224607&v=1.1.

## 3 Background

Premature ovarian insufficiency (POI) has experienced a notable surge in prevalence [1,2], affecting around 6 million Chinese women under 40 years, and emerges as a significant contributor to reproductive disorders and female infertility [3].

Conventional treatments such as hormone replacement therapy (HRT) and Fenmutong, while commonly employed, may yield unsatisfactory outcomes and are associated with notable side effects [4,5]. Consequently, a growing number of POI patients seek complementary and alternative medical (CAM) methods, including acupuncture, reflecting a quest for safer and potentially more effective interventions [6,7].

Acupuncture, a longstanding component of complementary and alternative medicine, has demonstrated promising effects in treating POI [8]. It activates the neural-endocrine-immune network, leading to increased beta-endorphin production, thus influencing the endocrine system and hormones. The appeal of acupuncture lies in its minimal side effects, safety, and cost-effectiveness, potentially addressing the root cause of the condition. Our research team has dedicated considerable effort to studying POI, with previous observations indicating that acupuncture not only alleviate perimenopausal symptoms, restore the menstrual cycle, and regulate serum hormone levels [[9], [10], [11], [12]]. Young patients with relatively good ovarian reserve function appear to experience more favorable treatment outcomes [13]. Conversely, older patients or those with relatively poor ovarian reserve function have also demonstrated improved efficacy with acupuncture therapy for POI, suggesting individual variability as a potential key factor influencing treatment outcomes [14,15]. Despite these observations, the underlying cause of individual response differences to acupuncture in women with POI remains unexplored.

Recent years have witnessed a rise in POI incidence, necessitating an in-depth investigation into its pathogenesis and treatment mechanisms. The advent of transcriptome sequencing technology in 1997 has revolutionized the study of POI's underlying mechanisms. Transcriptome sequencing not only serves as the foundation for identifying new POI-related genes but also facilitates the analysis of immune-related gene regulation, establishing correlations between different genes involved in POI pathogenesis. With approximately 90 genes currently linked to POI, its genetic heterogeneity becomes evident. For instance, studies exposing rats to environmental toxicants and utilizing transcriptome sequencing technology have identified DNA damage repair defects as pivotal in the pathogenesis of POI [[16], [17], [18]].

In this study, our primary objective is to gain a comprehensive understanding of the physiological significance of ovarian dysfunction in POI patients through peripheral blood transcriptome sequencing technology. The interactions between genes and ovarian function play a crucial role in elucidating the association between acupuncture action genes and individual differences observed in ovarian function recovery. To achieve these goals, we have designed a nested case-control study to evaluate the additional benefits of acupuncture treatment for women with POI. Additionally, we aim to investigate the mechanism underlying individual variations in response to acupuncture treatment, utilizing transcriptome gene sequencing analysis on peripheral blood samples. By employing this approach, we aim to systematically explore the potential mechanisms behind the observed individual curative effect differences with acupuncture in women with POI.

Through this research endeavor, we aspire to contribute valuable insights into the therapeutic effects of acupuncture for POI and shed light on the intricate interactions between genes and ovarian function. By understanding the underlying mechanisms, we hope to identify factors influencing the efficacy of acupuncture treatment in women with POI, paving the way for more personalized and effective treatment strategies for this condition. Ultimately, our study aims to contribute to the advancement of knowledge in the field of POI research, potentially improving the quality of life and outcomes for affected individuals.

## 4 Methods/design

### 4.1 Study design

The proposed investigation adopts an open-label, nested case-control study design to be conducted at Shenzhen Maternity and Child Healthcare Hospital. A total of 108 participants diagnosed with POI will be recruited for the study, and subsequent random assignment will be executed to allocate these patients into either the study group or the control group. The allocation ratio is set at 1:1, ensuring an equal distribution of 54 subjects in each group. Prior to the commencement of any intervention, explicit informed consent will be procured from all participating patients through the endorsement of a consent form administered by the researchers.

**Fig. 1:**
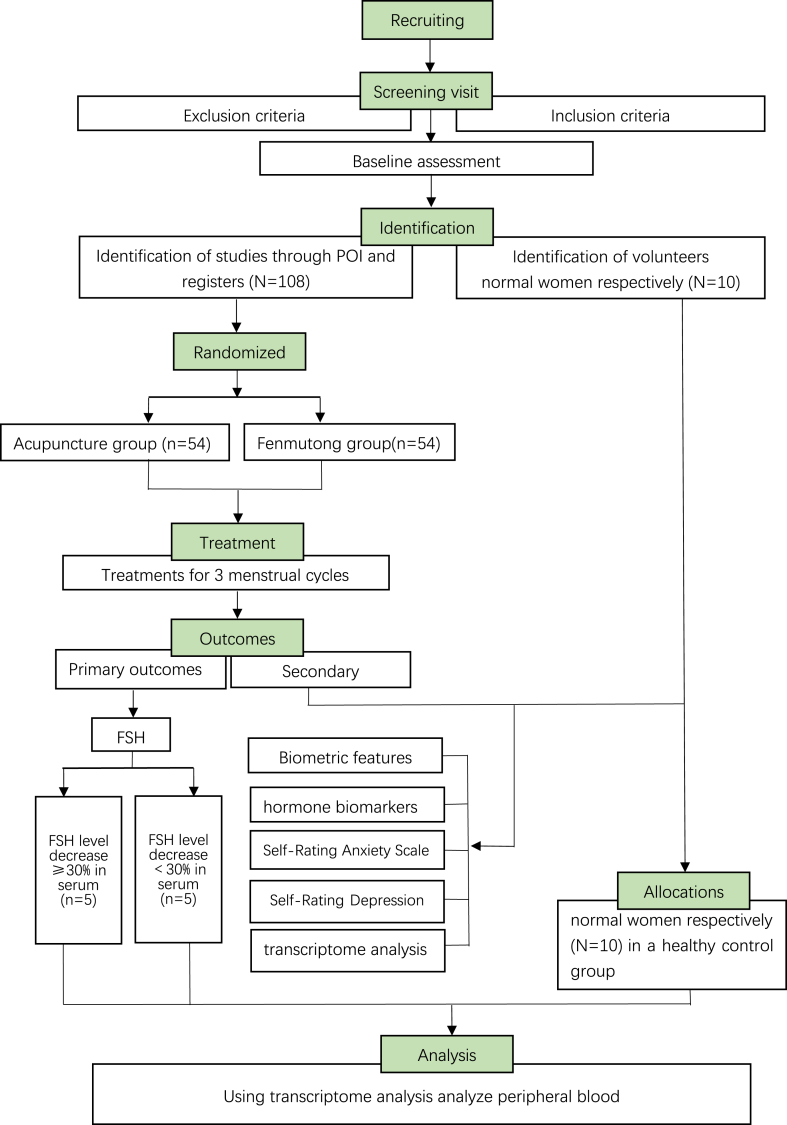
Study flow chart. Note: transcriptome analysis included 10 patients in the POI group and 10 patients in the Fenmutong group, with 5 patients with serum FSH levels ≥30 % and 5 patients with serum FSH levels <30 % in both groups.10 healthy women were selected as the control group.

Patients comprising the study group will exclusively undergo acupuncture treatment, while those in the control group will exclusively receive Fenmutong. To gain deeper insights into the mechanisms contributing to individual variations in the therapeutic outcomes of acupuncture, a subset of 10 patients from each group will undergo transcriptome analysis. This subset will encompass patients experiencing a serum FSH level decrease of ≥30 % post-acupuncture intervention, alongside those displaying a decrease of <30 % in serum FSH levels following the intervention. The trial's procedural flow is visually represented in Fig. 1, offering a comprehensive overview of the various stages and interventions integral to the study. Additionally, Table 1 provides meticulous details on the data collection process, delineating specific data points and measurements crucial for the comprehensive execution of the research.

**Table 1:** Overview of study visits.

Itern	Screening visit	Baseline visit	Treatment visit		End of treatment visit
			POI group	Control group	
Sign consent	×				
History	×				×
Biometric features	×	×			×
Transvaginal ultrasound	×	×	×	×	×
Routine test	×				×
hormone biomarkers		×			×
transcriptome analysis		×			×
Questionnaires		×			×
Acupuncture treatment			×		
Adverse events			×		

Note: Participants from the POI group will be selected for transcriptome analysis and will only be assessed at the initial screening visit. History, biometric features, routine test results, and transcriptome analysis will be assessed. Biometric features include body mass index (BMI); transvaginal ultrasound includes endometrial thickness, ovarian volume, antral follicle count, and size of ovarian cysts or developing follicles; Routine tests include blood routine examination, liver function, kidney function, routine stool, and urine tests; Hormone biomarkers include luteinizing hormone (FSH), follicle stimulating hormone (LH), total testosterone, progesterone, estradiol, and leptin; questionnaires include Self-Rating Anxiety Scale (SAS) and Self-Rating Depression Scale (SDS).

### 4.2 Paticipants recruitment

This study employs stringent inclusion and exclusion criteria to ensure the selection of participants aligns with the research objectives and mitigates potential confounding factors that could compromise the validity and results of the study.

### 4.3 Inclusion criteria

1 Participants must meet the diagnostic criteria for POI in accordance with the guidelines outlined in the 2016 edition of the European Society of Human Reproduction and Embryology (ESHRE) document titled " ESHRE Guideline: management of women with premature ovarian insufficiency." [19].
2 Eligible participants are women aged between 24 and 40 years.
3. Control subjects should have no history of reproductive disorders and should exhibit regular menstrual cycles, indicating normal ovarian function, and the Antral Follicle Count (AFC) should generally be between 5 and 12 per side [20].
4 Women in the POI group should possess Anti-Müllerian Hormone (AMH) values of less than 1.0 ng/ml.
5 The AFC should be less than 4 for women in the POI group.
6 All participants must consent to receive treatment according to the study program and willingly commit to being observed throughout the study.
7 Participants should provide informed consent, demonstrating comprehension of the study procedures, potential risks and benefits, and their voluntary willingness to participate.
8 Participants must willingly register their case for the study.

Meeting these inclusion criteria ensures the selection of participants capable of contributing valuable data to effectively achieve the research objectives.

### 4.4 Exclusion criteria

1 Women who are currently pregnant or experiencing menopause at the time of consultation.
2 Individuals with confirmed organic lesions causing POI through conventional gynecological examinations, including imaging and secretion examinations.
3 Participants who have undergone relevant treatments that may potentially influence the observation indicators or treatment outcomes.
4 Individuals with severe heart, liver, and kidney injuries that may impact normal metabolism.
5 Participants with combinations of other physiological or pathological conditions that may affect the study's outcome indicators.
6 Those who withdraw from treatment due to personal reasons.
7 Participants who fail to strictly adhere to the intervention measures, leading to the inability to collect relevant data or the production of errors in feedback on efficacy and effect indicators.
8 Individuals experiencing severe adverse reactions or complications during treatment, rendering them unable to continue the intervention.
9 Participants using drugs that may interfere with the study's treatment plan.
10 Individuals simultaneously participating in other experiments.
11 Participants unable to complete the filling of various scales.
12 Individuals unwilling to be randomly grouped.
13 Patients with abnormal thyroid function.

Application of these exclusion criteria aims to ensure the suitability of selected participants for the research objectives and reduce potential confounding factors.

### 4.5 Intervention

Informed consent shall be diligently obtained from all participants, elucidating the potential advantages associated with embracing a health-conscious lifestyle, encompassing regular exercise and a nutritionally balanced diet. The study's intervention will be bifurcated into distinct groups: the study group, exclusively undergoing acupuncture treatment, and the control group, solely subjected to Fenmutong administration. The intervention duration for both groups is stipulated at three menstrual cycles. The administration of acupuncture treatments for the study group will be executed by adept professional acupuncturists possessing a minimum of 2 years of practical experience in acupuncture techniques.

### 4.6 Acupuncture treatment

This study is conducted in accordance with the Standards for Reporting Interventions in Clinical Trials of Acupuncture (STRICTA) guidelines [21], thereby ensuring a comprehensive and transparent approach to acupuncture treatment for POI. The selection of acupoints is based on the Zang-fu organ system, the Yin-Yang theory, and clinical guidelines, with two sets of acupoints alternately applied. Sterilised, single-use needles from the Huatuo Suzhou Medical Appliance Factory are employed following the disinfection of the skin. The methods of needle manipulation for each acupoint featured in this study are presented in Table 2 in meticulous detail. The STRICTA checklist, which details the study's compliance with these standards, is included as supplementary material. Each acupuncture session, with a standard duration of 30 min and conducted three times a week, is designed to achieve de qi, a sensation integral to acupuncture efficacy. The treatment regimen is meticulously documented in accordance with the STRICTA guidelines, from the anatomical specifics and needle manipulation techniques to the consistency across all sessions, spanning up to three menstrual cycles.

**Table 2:** Acupuncture protocol.

Acupoints	Location	Method
Set 1		
CV-4 (Guanyuan)	On the anterior median line of the lower abdomen,3cun below the navel	1.0–1.5cun perpendicularly insertion
EX-CA1 (Zigong)	On the lower abdomen, 4cun below the navel, 3cun lateral to Zhongji (RN-3)	1.0–1.5cun perpendicularly insertion
ST-29 (GuiLai)	On the lower abdomen,4 cun below the umbilicus,and 2 cun lateral to the anterior midline.	1.0–1.5cun perpendicularly insertion
CV-3 (Zhongji)	On the anterior median line of the lower abdomen,4cun below the navel	1.0–1.5cun perpendicularly insertion
RN-12 (Zhongwan)	On the anterior median line of the upper abdomen, 4cun below the navel	1.0–1.5cun perpendicularly insertion
ST-36 (Zusanli)	3 cun directly below Dubi,and one finger-breadth lateral to the anterior border of the tibia.	1.0–1.5cun perpendicularly insertion
SP-10 (Xuehai)	2 cun above the upper border of the medial patella	1.0–1.5cun perpendicularly insertion
LR-3 (Taichong)	On the dorsum of the foot, in the depression proximal to the first metatarsal space	1.0–1.5cun perpendicularly insertion
Set2		
BL-18 (Ganshu)	On the back, under the spinous process of the 9th thoracic vertebra, 1.5 cun laterally	0.5–0.8cun diagonally insertion
BL-20 (Pishu)	On the back, under the spinous process of the 11th thoracic vertebra,1.5 cun laterally	0.5–0.8cun diagonally insertion
BL-23 (Shenshu)	On the back, under the spinous process of the 2nd lumbar spine,1.5 cun laterally	0.5–0.8cun diagonally insertion
BI-26 (Guanyuanshu)	On the back, under the spinous process of the 5th lumbar spine,1.5 cun laterally	0.5–1.0cun diagonally insertion
BL-32 (Ciliao)	In the region of the sacrum, between the posterior superior iliac spine and the posterior median line, in the 2nd posterior sacral foramen	2.5–3.0cun oblique insertion to the 2nd posterior sacral foramen
SP-6 (Sanyinjiao)	On the medial side of the shank, 3cun above the medial malleolus, by the posterior of the tibia	1.0–1.5cun perpendicularly insertion
KI-3 (Taixi)	On the medial side of the foot, posterior to the medial malleolus, in the depression between the tip of the medial malleolus and the tendon calcaneus	0.5–1.0cun perpendicularly insertion

Note: Two sets of acupoints will be used; each set will be used on alternate treatments.

### 4.7 Fenmutong administration

The administration of Fenmutong treatment commences on the fifth day of menstruation specifically for participants diagnosed with POI assigned to the control groups. Initially, participants undertake oral administration of 2 mg estradiol tablets (brick-red tablets) daily for a consecutive period of 14 days. Subsequently, they transition to the oral intake of estradiol 2 mg + dydrogesterone 10 mg (yellow tablets) for another 14 consecutive days. A single course of this treatment spans 28 days. In the event of menstruation occurring during the medication period, the medication is recommenced from the 5th day of the subsequent menstrual cycle. If menstruation does not transpire, the subsequent course is initiated on the 7th day after the cessation of medication. The entirety of the treatment protocol extends across three menstrual cycles.

For participants experiencing amenorrhea at the time of consultation, immediate initiation of medication on the same day is permissible. Conversely, participants with infrequent menstruation should commence medication on the 5th day of the menstrual cycle, following the previously outlined methodology.

## 5 Outcome measures

### 5.1 Primary outcome: change in FSH levels

The principal outcome measure in this study revolves around the alteration observed in FSH levels. This metric serves as a pivotal gauge to evaluate the effectiveness of acupuncture treatment when employed as an adjunctive therapy for POI. The data collection process is bifurcated into two distinct time points: the baseline assessment conducted prior to the study's initiation and a subsequent assessment following the culmination of three menstrual cycles of acupuncture treatments. This meticulous approach aims to provide comprehensive insights into the impact of acupuncture on FSH levels over the specified duration, thereby contributing to the overall assessment of its therapeutic efficacy for POI.

### 5.2 Secondary outcomes

The principal outcome measure in this study revolves around the alteration observed in FSH levels. This metric serves as a pivotal gauge to evaluate the effectiveness of acupuncture treatment when employed as an adjunctive therapy for POI. The data collection process is bifurcated into two distinct time points: the baseline assessment conducted prior to the study's initiation and a subsequent assessment following the culmination of three menstrual cycles of acupuncture treatments. This meticulous approach aims to provide comprehensive insights into the impact of acupuncture on FSH levels over the specified duration, thereby contributing to the overall assessment of its therapeutic efficacy for POI. In conjunction with the primary focus on FSH levels, this study encompasses an exploration of secondary outcomes to foster a holistic comprehension of acupuncture's impact on POI. Secondary outcome measures encompass biometric features, hormone biomarkers, Self-Rating Anxiety Scale, Self-Rating Depression Scale, and transcriptome analysis. The data acquisition for these secondary outcomes is structured around two pivotal time points: the baseline assessment before study commencement and the post-intervention assessment after the conclusion of acupuncture sessions.

Comparative analysis of these datasets enables researchers to discern alterations in various parameters resultant from acupuncture treatment. Specifically, to scrutinize the distinct genes associated with individual variations in acupuncture's therapeutic effects, gene abundance profiling will be conducted within the study group and juxtaposed with the control group. Transcriptome analysis, performed on peripheral blood samples from 5 participants in each group exhibiting a ≥30 % reduction or <30 % reduction in serum FSH levels post-intervention, aims to unravel the molecular intricacies underlying acupuncture's differential efficacy. An additional 10 subjects, meeting the study criteria, will be enlisted for the healthy control group. The ensuing data analysis will be executed utilizing SPSS 25.0 for comprehensive evaluation.

## 6 Transcriptome analysis

### 6.1 RNA extraction, library preparation and sequencing

The extraction of RNA commenced with the utilization of TRIzol reagent (Invitrogen, Carlsbad, CA, USA), conducted in strict adherence to the manufacturer's stipulated procedures. Subsequent to extraction, the Nanodrop instrument facilitated an evaluation of the quality and concentration of the obtained RNA. Additionally, the Agilent 2100 Bioanalyzer (Agilent, CA, USA) was employed to assess RNA integrity, with only samples exhibiting a RNA integrity number (RIN) surpassing 7 deemed suitable for subsequent RNA library constitution and ensuing analyses.

Library construction transpired through the application of the Illumina TruSeq Stranded mRNA Library Prep Kit (Illumina, CA, USA), aligning with the manufacturer's directives. This process initiated by enriching mRNA molecules with polyA tails through the use of magnetic beads, effectively eliminating ribosomal RNA (rRNA) and other non-coding RNA species. Subsequent steps included reverse transcription employing random hexamer primers for synthesizing the first strand of cDNA, succeeded by DNA polymerase-mediated synthesis of the second cDNA, ultimately generating double-stranded cDNA molecules. The ensuing steps involved end repair and A-tailing, followed by the ligation of Illumina TruSeq index adapters. To enhance the library, PCR amplification was executed, thereby augmenting the quantity of cDNA fragments available for sequencing. The thorough assessment of library quality and size distribution was conducted using the Agilent 2100 Bioanalyzer.

Sequencing procedures were carried out on the Illumina NovoSeq 6000 platform, employing paired-end 150 bp reads. The resultant sequencing data, formatted in FASTQ, underwent quality control via the FastQC software.

### 6.2 Bioinformatic and statistical analysis

Quantification of RNA sequencing data was executed utilizing the kallisto software [22]. Differential expression analysis was conducted with the DESeq2 package [23] to identify genes with distinct expression patterns. Additionally, the cluster Profiler package [24]

was employed to enhance the analysis of differentially expressed genes.

Continuous variables' normality was assessed using the Shapiro-Wilk test in this study. For normally distributed continuous variables, data were presented as mean ± SD (standard deviation), and intergroup comparisons utilized Student's t-test. Conversely, skewed continuous variables were expressed as median (interquartile range, IQR) and compared through the nonparametric Wilcoxon rank-sum test. Categorical variables were reported as frequencies (percentages, %) and subjected to comparison via the chi-square test.

To explore associations between gene abundance and various clinical indicators pertaining to ovarian reserve, ovarian endocrine function, and perimenopausal syndrome symptoms, Pearson's correlation analysis was employed. For genes exhibiting significant differences, receiver operating characteristic (ROC) curves were constructed to assess their diagnostic performance. The area under the curve (AUC) value served as a metric for discriminatory power. Statistical significance was set at a two-tailed *P* < 0.05. R software was utilized for all data analyses.

### 6.3 Adverse events

The emphasis on safety in this clinical trial is paramount, particularly concerning acupuncture interventions. Adverse events related to acupuncture primarily involve local ecchymoses, fainting, significant pain, and local infections. These events are closely tied to patients' subjective experiences and the proficiency of acupuncturists in their needling techniques. To ensure a secure operation throughout the trial, all participating acupuncturists will undergo comprehensive pretrial training and must successfully pass training and assessment to ensure uniform standards.

Throughout the treatment process, any adverse events stemming from acupuncture will be meticulously recorded. Operators will respond promptly and appropriately, implementing necessary measures to address adverse events as they arise. In cases where serious adverse events occur, investigators will take immediate action to suspend the clinical trial. Swift administration of effective treatments will follow to manage and mitigate the severity of the event. Furthermore, any occurrence of serious adverse events will be promptly reported to the ethical review board at the Shenzhen Maternity and Child Healthcare Hospital within 24 h. The entire process, encompassing details of adverse events and their management, will be meticulously documented for comprehensive record-keeping and subsequent analysis.

### 6.4 Sample size

The calculation of the sample size is derived from the formula comparing two sample rates：

$$N=\frac{{\left(Z_{1-\alpha \;}+Z_{1-\beta }\right)}^{2}\left(1+\frac{1}{k}\right)\cdot \sigma^{2}}{{\left(\mu_{T\;}-\mu_{C}-△\right)}^{2}})$$

Here, N represents the required sample size for each group, Pe and *Pc* denote the maximum and minimum effective rates of each group. Based on our retrospective clinical data and previous experimental reports, acupuncture demonstrated a reduction in FSH levels by 20 IU/L, while assuming that the control group reduced FSH levels by 14.5 IU/L. For the study, which is divided into two groups with parameters $\mu_{T\;}$ = 20, $\mu_{C}$ = 14.5, △=3, a test level α = 0.05 and a test efficiency 1-β = 0.9 were considered.

In light of an anticipated 20 % dropout rate, a total of 108 patients will be recruited, ensuring no less than 54 participants in each group. Given the absence of a standardized sample size calculation for transcriptome analysis, the approach aligns with prevalent studies on POI. From the pool of 108 participants, 10 patients in each group (comprising 5 with a decrease of ≥30 % in serum FSH levels after intervention, 5 with a decrease of <30 % in serum FSH levels after intervention, and 10 normal female volunteers in the healthy control group) will be selected for transcriptome analysis.

### 6.5 Allocation and blinding

Participant allocation to either the study or control group will follow a meticulous 1:1 ratio, determined by adherence to predetermined inclusion and exclusion criteria. A prospective cohort study protocol has been devised for individuals diagnosed with POI, stratified by the extent of FSH decline. The exposure factor under investigation is acupuncture treatment, with categorization based on FSH decline exceeding 30 % or less than 30 %, comprising 5 cases each.

In this openly conducted clinical trial, recognition is accorded to the inherent awareness of both patients and involved acupuncturists regarding treatment assignments due to the study's nature. However, to counterbalance potential biases and uphold the scientific rigor of the investigation, specific measures will be instituted to ensure the maintenance of blinding for other pivotal personnel involved in the study.

### 6.6 Data collection and management

Data collection will be conducted at two critical junctures: baseline and postintervention. Trained data collectors, employing case report forms (CRFs), will meticulously record participant information. Specifically, the anxiety and depression levels of participants will be assessed through the administration of the Self-Rating Anxiety Scale (SAS) and Self-Rating Depression Scale (SDS), respectively. Following the CRF-based data collection, the information will be meticulously entered into an electronic database to facilitate streamlined data management.

To bolster accuracy, a dual data entry system will be implemented, involving two data collectors to independently input the data, thereby minimizing errors. Subsequently, the clinical raw data will undergo scrutiny by the project supervisor, who will rectify any identified errors or inconsistencies before proceeding with data export. Once the data's accuracy is affirmed, the database will be securely locked, paving the way for subsequent statistical analyses. Participant identification will be maintained through assigned numbers, and the database will be password-protected to uphold confidentiality.

Oversight of the data will be conducted by the ethical review board of the Shenzhen Maternity and Child Healthcare Hospital. Comprehensive reporting on participant withdrawals and the reasons for withdrawal will be undertaken. In the event of dropouts, modern imputation methods for handling missing data will be employed, ensuring their inclusion in the subsequent analyses.

### 6.7 Statistical methods

Data analysis will be executed utilizing SPSS version 25.0 (SPSS Inc., Chicago, IL, USA) by two qualified statisticians, ensuring the attainment of robust and reliable results.Descriptive statistics, comprising mean, median, 25th percentile, 75th percentile, standard deviation, maximum values, and minimum values, will be employed to present the results of the measurement data analysis. Group comparisons will be facilitated using either the independent sample *t*-test or the Mann-Whitney U rank sum test, contingent upon the normal distribution and homogeneous variance of the measurement data. Enumeration data will be characterized by frequency and constituent ratios, with group comparisons executed through Pearson's χ2 test or Fisher's exact test. Correlations between different indicators will be explored through Pearson and Spearman correlations. A significance threshold of P < 0.05, employing a two-sided test, will dictate statistical significance. Additionally, metagenomic analysis of the gut microbiome will be conducted by BGI Shenzhen Co., Ltd (China).

### 6.8 Patient and public involvement

Patients were not involved in the design and conduct of the study. Participants will be able to view the study results via social media.

## 7 Discussion

Acupuncture, rooted in traditional Chinese medicine and boasting a millennia-long history, has gained global recognition as a therapeutic modality for diverse medical conditions [25]. Integral to Chinese medicine, acupuncture stands out for its non-invasive nature, convenience, and minimal adverse effects, rendering it applicable in the prevention, treatment, and rehabilitation of numerous ailments [26]. Substantial evidence from both clinical and laboratory studies has highlighted acupuncture's efficacy in addressing inflammation, immune responses, and various chronic gynecological and endocrine disorders [[27], [28], [29]]. Recent years have witnessed significant progress in clinical and experimental research, particularly in the context of POI [12,13,28]. Acupuncture demonstrates distinct advantages in mitigating clinical symptoms of POI, regulating sex hormone levels, revitalizing residual follicles, and restoring menstrual cycles [8,[30], [31], [32]]. Despite these advancements, the precise mechanisms underpinning acupuncture's impact on follicular revival and hormone regulation demand further exploration.

Given the escalating incidence of POI, understanding acupuncture's potential to impede its progression assumes paramount importance [33]. While debates persist regarding the scientific substantiation of acupuncture's benefits, it exhibits promise in treating infertility disorders associated with POI and sex hormone imbalances [34,33]. The advent of RNA Seq technology has become instrumental in unraveling the molecular-level mechanisms of acupuncture [35,36]. Nevertheless, scant attention has been devoted to discerning the individualized effects of acupuncture on POI patients, and the potential inter-individual variations in efficacy remain largely unexplored. Furthermore, the transcriptomic landscape of acupuncture treatment for POI remains a largely uncharted territory.

To address these knowledge gaps, we propose a nested case-control study employing transcriptome sequencing technology to analyze peripheral blood samples from POI patients subjected to acupuncture [37,38]. This approach seeks to elucidate the potential mechanisms governing individual differences in acupuncture efficacy for POI. By scrutinizing transcriptomic changes associated with acupuncture treatment, the study endeavors to evaluate the efficacy of acupuncture for POI and contribute valuable evidence-based insights [[39], [40], [41]]. Ultimately, it aspires to furnish a robust clinical and experimental foundation, paving the way for further investigations into acupuncture's therapeutic potential in POI.

However, it is crucial to acknowledge several limitations accompanying this study. The utilization of serum samples may introduce confounding factors, as local tissue samples might offer greater sensitivity to interference. Despite stringent inclusion and exclusion criteria, the study's results may not comprehensively capture the nuanced mechanisms underlying individual differences in acupuncture efficacy. Additionally, the absence of a sham acupuncture group as a control limits our ability to rule out potential placebo effects influencing observed outcomes. Lastly, the relatively modest sample size employed for transcriptome analysis, although justified considering cost constraints and typical sample sizes in such studies, underscores the challenges inherent in determining optimal sample sizes for transcriptomic investigations.

## Trial status

The protocol version number is 20220725, V3.0 (2022/07/25). This study has started in March 2023, and the recruitment is expected to be completed in 2024.

## Ethics approval and consent to participate

This study was performed in line with the principles of the Declaration of Helsinki (2013 version) and was approved by the Ethics Committee of Shenzhen Maternity and Child Healthcare Hospital (No [2022]058), Shenzhen, China. All participants will fill in informed consents and written informed consent will be obtained from all the participants prior to enrolment.

## Consent for publication

Not applicable.

## Availability of data and materials

Data and materials can be obtained from the corresponding author after the trial.

## Funding

This work was supported by Guangdong Province Key Field R&D Plan (2020B1111100003); 10.13039/501100004791Shenzhen Science and Innovation Commission (SZSTIC) (No. JCYJ20210324130001004); Sanming Project of Medicine in Shenzhen (SZZYSM202311010). We would like to thank all the participants of this study for their cooperation and patience.

## CRediT authorship contribution statement

**Shiyu Feng:** Writing – original draft, Data curation. **Yu Luo:** Methodology, Data curation. **Yan Chen:** Resources, Investigation. **Haimin Zhu:** Data curation, Conceptualization. **Tianqi Zhao:** Methodology, Investigation. **Fei Ma:** Methodology. **Yanting Lin:** Software, Resources. **Yan Ning:** Project administration, Funding acquisition. **Jiaman Wu:** Writing – review & editing, Supervision, Project administration, Funding acquisition, Data curation.

## Declaration of competing interest

The authors declare that the research was conducted in the absence of any commercial or financial relationships that could be construed as a potential conflict of interest.

## Abbreviations

POI: premature ovarian insufficiency
FSH: Follicle-Stimulating Hormone
LH: luteinizing hormone
HRT: hormone replacement therapy
E2: estrogen
ESHRE: European Society of Human Reproduction and Embryology
AMH: anti-Müllerian hormone
AFC: antral follicle counts
CRF: case report forms
FAS: Full analysis set
PPS: per-protocol analysis set
SS: safety set
ITT: intention-to-treat
SAS: Self-Rating Anxiety Scale
SDS: Self-Rating Depression Scale
CAM: Complementary and alternative medical
