# Supplementary material for: Individualized responses to acupuncture in premature ovarian insufficiency: A study protocol for a nested case-control trial with transcriptome analysis
Source: Heliyon. 2024 Sep 12;10(18):e37859. doi: 10.1016/j.heliyon.2024.e37859 (PMC11425121; doi:10.1016/j.heliyon.2024.e37859)
Supplement: Multimedia component 1 [file mmc1.pdf]

ChiCTR2300068981 版本V1.1 版本创建时间2023/05/17 11:12:38 中国临床试验注册中心

审核状态：通过审核
Project audit state：Successful

注册号：
Registration number：ChiCTR2300068981

最近更新日期：
Date of Last Refreshed on：2023-03-03

注册时间：
Date of Registration：2023-03-03

注册号状态：预注册

Registration Status：Prospective registration

注册题目：基于转录组测序技术对针刺治疗早发性卵巢功能不全的机制研究

Public title：Study on the mechanism of acupuncture treatment of premature ovarian insufficiency based on transcriptome sequencing technology

注册题目简写：

English Acronym：

研究课题的正式科学名称：基于转录组测序技术对针刺治疗早发性卵巢功能不全的机制研究

Scientific title：Study on the mechanism of acupuncture treatment of premature ovarian insufficiency based on transcriptome sequencing technology

研究课题代号(代码)：
Study subject ID：

在二级注册机构或其它机构的注册号：
The registration number of the Partner Registry or other register：

申请注册联系人：	吴家满	研究负责人：	吴家满
Applicant：	Jiaman WU	Study leader：	Jiaman WU
申请注册联系人电话：<br>Applicant telephone：	15817470407	研究负责人电话：<br>Study leader's telephone：	15817470407
申请注册联系人传真：<br>Applicant Fax：		研究负责人传真：<br>Study leader's fax：	
申请注册联系人电子邮件：<br>Applicant E-mail：	wujiaman202@163.com	研究负责人电子邮件：<br>Study leader's E-mail：	wujiaman202@163.com
申请单位网址(自愿提供)：<br>Applicant website(voluntary supply)：		研究负责人网址(自愿提供)：<br>Study leader's website(voluntary supply)：	
申请注册联系人通讯地址：	广东省深圳市福田区福强路3012号	研究负责人通讯地址：	广东省深圳市福田区福强路3012号
Applicant address：	No.3012 Fuqiang Road, Futian District, Shenzhen, Guangdong, China	Study leader's address：	No.3012 Fuqiang Road, Futian District, Shenzhen, Guangdong, China
申请注册联系人邮政编码：<br>Applicant postcode：		研究负责人邮政编码：<br>Study leader's postcode：	

申请人所在单位：深圳市妇幼保健院

Applicant's institution：Shenzhen Maternity and Child Healthcare Hospital, Southern Medical University

研究负责人所在单位：深圳市妇幼保健院

Affiliation of the Leader：Shenzhen Maternity and Child Healthcare Hospital, Southern Medical University

---

是否获伦理委员会批准：是

Approved by ethic committee：Yes

伦理委员会批件文号：<br>Approved No. of ethic committee：	SFYLS[2022]058	伦理委员会批件附件：<br>Approved file of Ethical Committee：	查看附件View

批准本研究的伦理委员会名称：深圳市妇幼保健院

Name of the ethic committee：Shenzhen Maternity and Child Healthcare Hospital

伦理委员会批准日期：
Date of approved by ethic committee：2022-10-27

伦理委员会联系人：戴宇婷

Contact Name of the ethic committee：Dai Yuting

伦理委员会联系地址：广东省深圳市福田区红荔路2004号

Contact Address of the ethic committee：No.2004 Hongli Road, Futian District, Shenzhen, Guangdong, China

伦理委员会联系人电话：<br>Contact phone of the ethic committee：	+86 755 82869849	伦理委员会联系人邮箱：<br>Contact email of the ethic committee：	

研究实施负责（组长）单位：深圳市妇幼保健院

Primary sponsor：Shenzhen Maternity and Child Healthcare Hospital

研究实施负责（组长）单位地址：广东省深圳市福田区红荔路路2004号

Primary sponsor's address：No.2004 Hongli Road, Futian District, Shenzhen, Guangdong, China

试验主办单位(项目批准或申办者)：
Secondary sponsor：

国家：	中国	省(直辖市)：	广东省	市(区县)：	深圳市
Country：	China	Province：	Guangdong	City：	Shenzhen
单位(医院)：	深圳市妇幼保健院	具体地址：	广东省深圳市福田区红荔路路2004号		
Institution hospital：	Shenzhen Maternity and Child Healthcare Hospital	Address：	No.2004 Hongli Road, Futian District, Shenzhen, Guangdong, China		

经费或物资来源：2021深圳市科技计划项目基础研究（JCYJ20210324130001004）

Source(s) of funding：Shenzhen Technology and Innovation Committee (Natural Science Foundation) (No. JCYJ20210324130001004)

研究疾病：早发性卵巢功能不全

Target disease：premature ovarian insufficiency

研究疾病代码：

Target disease code：

研究类型：干预性研究

Study type：Interventional study

研究所处阶段：I期临床试验

Study phase：1

研究设计：巢式病例-对照研究

Study design：Nested case-control study

研究目的：采用巢式病例-对照的方式，招募早发性卵巢功能不全（POI）患者，进行针灸临床诊疗、疗效及安全性的信息采集，POI患者针刺治疗前后的血液标本，通过对比针刺前后标本中mRNA的差异，进而观察转录最终产物的变化，评价针刺治疗POI患者卵巢功能的有效性和安全性。

Objectives of Study：The nested case-control method was used to recruit patients withpremature ovarian insufficiency (POI) and collect information on the clinical diagnosis, treatment, efficacy and safety of acupuncture. The blood samples of POI patients before and after acupuncture treatment were compared with the mRNA differences in the samples before and after acupuncture, and then the changes in the final transcript products were observed to evaluate the effectiveness and safety of acupuncture treatment of ovarian function in POI patients.

药物成份或治疗方案详述：

Description for medicine or protocol of treatment in detail：

纳入标准：1.参考 Guideline of the European Society of Human Reproduction and Embryology符合西医早发性卵巢功能不全诊断； 2.年龄＜40岁的育龄妇女； 3.月经稀发或闭经 ≥4个月； 4.FSH>25IU/L（至少２次检查，２次检查间隔时间１个月以上）； 5.同意接受按本方案治疗及追踪观察者； 6.愿意签署知情同意书； 7.愿意进行病例注册。

Inclusion criteria：1.Reference to the Guideline of the European Society of Human Reproduction and Embryology is consistent with the diagnosis of premature ovarian insufficiency in western medicine; 2. Women of childbearing age less than 40 years old;3. Menstrual rareness or amenorrhea ≥ 4 months;4. FSH>25IU/L (at least 2 inspections, and the interval between two inspections is more than 1 month);5. Agreed to receive treatment according to this program and follow up observers; 6. Willing to sign informed consent; 7. Willing to register a case.

排除标准：①卵巢切除患者；② 有放疗或化疗史；③ 有尿道感染或肿瘤；④ 有自身免疫性疾病；⑤ 有先天生殖器官异常；⑥合并有心血管、肾、肝和造血系统等严重原发性疾病；⑥ 智力行为障碍，不能配合完成临床观察者；

Exclusion criteria：① Ovariectomy patients;② Have a history of radiotherapy or chemotherapy;③ Urethral infection or tumor;④ Have autoimmune diseases;⑤ Have congenital reproductive organ abnormality;⑥ Complicated with serious primary diseases such as cardiovascular, kidney, liver and hematopoietic system;⑥ Mental and behavioral disorders, unable to cooperate in clinical observation;

研究实施时间：<br>Study execute time：	从 From 2023-02-28至 To 2024-02-29	征募观察对象时间：<br>Recruiting time：	从 From 2023-03-03 至 To 2023-08-31

干预措施：
Interventions：

组别：	试验组	样本量：	30
Group：	Experimental Group	Sample size：	
干预措施：	针刺	干预措施代码：	
Intervention：	Acupuncture	Intervention code：	
组别：	对照组	样本量：	30
Group：	control Group	Sample size：	
干预措施：	不予干预	干预措施代码：	
Intervention：	No intervention	Intervention code：	

研究实施地点：
Countries of recruitment and research settings：

国家：	中国	省(直辖市)：	广东	市(区县)：	深圳
Country：	China	Province：	Guangdong	City：	Shenzhen
单位(医院)：	深圳市妇幼保健院	单位级别：	三级甲等		
Institution hospital：	Shenzhen Maternity and Child Healthcare Hospital	Level of the institution：	Tertiary A		

测量指标：
Outcomes：

指标中文名：	性激素	指标类型：	主要指标
Outcome：	sex hormone	Type：	Primary indicator
测量时间点：	月经第2-5天	测量方法：	血清
Measure time point of outcome：	2-5 days after menstruation	Measure method：	serum
指标中文名：	转录组测序	指标类型：	次要指标
Outcome：	Transcriptome sequencing	Type：	Secondary indicator
测量时间点：	月经第2-5天	测量方法：	血清
Measure time point of outcome：	2-5 days after menstruation	Measure method：	serum
指标中文名：	抗苗勒氏管激素	指标类型：	主要指标
Outcome：	AMH	Type：	Primary indicator
测量时间点：	月经第2-5天	测量方法：	血清
Measure time point of outcome：	2-5 days after menstruation	Measure method：	serum

---

采集人体标本：
Collecting sample(s) from participants：

标本中文名：	血清	组织：	
Sample Name：	Blood	Tissue：	
人体标本去向	使用后销毁	说明	
Fate of sample：	Destruction after use	Note：	

征募研究对象情况：<br>Recruiting status：	尚未开始<br>Not yet recruiting	年龄范围：<br>Participant age：	最小 Min age 19 岁 years<br>最大 Max age 39 岁 years
性别：	女性	Gender：	Female

随机方法（请说明由何人用什么方法产生随机序列）：由专人将全部入选患者按就诊顺序编号，采用SAS软件设定种子数（seed）进行随机化分配，依次装入不透明信封中，由专人保管。在分组时保证各组患者年龄、性别、病程等均衡性。

Randomization Procedure (please state who generates the random number sequence and by what method)：All the selected patients were numbered by special personnel according to the order of treatment, and the seed number (seed) was set by SAS software for random allocation, which was put into an opaque envelope one by one and kept by special personnel.The balance of age, sex and course of disease

---

是否公开试验完成后的统计结果：
Calculated Results after the Study Completed public access：公开/Public

---

盲法：Not stated

Blinding：Not stated

试验完成后的统计结果（上传文件）：点击下载

Calculated Results after the Study Completed(upload file)：download

---

是否共享原始数据：
IPD sharing：是Yes

共享原始数据的方式（说明：请填入公开原始数据日期和方式，如采用网络平台，需填该网络平台名称和网址）：以论文的形式发表

The way of sharing IPD"(include metadata and protocol, If use web-based public database, please provide the url)：Published as a thesis

数据采集和管理（说明：数据采集和管理由两部分组成，一为病例记录表(Case Record Form, CRF)，二为电子采集和管理系统(Electronic Data Capture, EDC)，如ResMan即为一种基于互联网的EDC：病例记录表

Data collection and Management (A standard data collection and management system include a CRF and an electronic data capture：Case sheet

数据与安全监察委员会：
Data and Safety Monitoring Committee：无/No
